# The effects of prediction errors and movement speed on pseudo-haptic sensations

**DOI:** 10.3389/fpsyg.2026.1774826

**Published:** 2026-03-30

**Authors:** Yosuke Suzuishi, Shusaku Shinada, Acer Yu-Chan Chang, Wen Wen

**Affiliations:** 1 Department of Psychology, Rikkyo University, Niiza-shi, Japan; 2 Helsinki Collegium for Advanced Studies, University of Helsinki, Helsinki, Finland; 3 Department of Computer Science, University of Helsinki, Helsinki, Finland

**Keywords:** illusion, prediction error, pseudo-haptic sensations, sense of agency, touch, visuo-tactile interaction

## Abstract

Pseudo-haptic sensations are illusory haptic experiences induced by manipulating the visual feedback of object movements. Previous studies have suggested that such sensations arise from prediction errors generated by a forward model, defined as the discrepancy between predicted and actual outcomes. In contrast, other studies propose that pseudo-haptic sensations emerge from a simple statistical relationship between weight and movement speed (e.g., heavier objects move more slowly), with prediction errors being only a by-product. In the present study, we further investigated the mechanisms underlying pseudo-haptic sensations by separating spatial prediction errors from movement speed. Participants moved a cursor along a sine-wave-shaped pathway using a mouse. The cursor's movement direction was generated by combining participants' real-time mouse movements with pre-recorded movements from another individual, producing three levels of control. The cursor's movement speed remained proportional to the mouse movement. On the other hand, when the cursor entered a central painted zone on the screen, its speed was reduced by three different ratios while remaining proportional to the mouse movement. Participants rated their sense of resistance in the hand and their sense of agency over the cursor's movement. A linear mixed-effects model analysis, with spatial prediction error and the time required to reach the end of the pathway as fixed effects and participant as a random effect, showed that both spatial prediction error and effective movement speed (indexed by trial completion time) significantly contributed to the sense of resistance. Taken together, these findings suggest that both prediction errors and object movement speed contribute to pseudo-haptic sensations, likely through different processes.

## 1 Introduction

In daily life, we perceive sensory information through our eyes, ears, skin, and other sensory receptors, forming perceptions within each modality. However, these sensory modalities are not independent of one another (Shimojo and Shams, 2001) and are closely linked to our actions. Research on multimodal interactions has shown that perceptual experiences are constructed from multisensory information (Calvert et al., 2004; Stein, 2012), enabling robust and coherent representations of the external world (Ernst and Bülthoff, 2004). Nevertheless, multimodal interactions do not always make perception more precise. In some cases, input from one sensory modality can modulate or alter another in ways that deviate from the actual physical properties of the external world. A representative example is the phenomenon of pseudo-haptic sensations, in which visual feedback that is distorted relative to an observer's input induces illusory haptic experiences such as weight (Dominjon et al., 2005; Samad et al., 2019), compliance (Lecuyer et al., 2000), or friction (Lecuyer et al., 2000; Ujitoko et al., 2019), even in the absence of actual haptic stimulation (Ujitoko and Ban, 2021) for a review.

Pseudo-haptic sensations are typically induced by manipulating the control/display (C/D) ratio, where *control* refers to the magnitude of the observer's input and *display* refers to the magnitude of the visual feedback. For example, in studies of weight perception (Dominjon et al., 2005; Samad et al., 2019), participants physically held an object (i.e., control) while viewing only its virtual representation (i.e., display). When the C/D ratio was smaller than 1—that is, when the physical displacement of the object (control) was amplified in the virtual representation (display)—participants perceived the object as lighter. Conversely, as the C/D ratio increased, the object was perceived as heavier. In addition to manipulations of the C/D ratio, delays in the response of a controlled object can also induce pseudo-haptic sensations such as heaviness or resistance when participants control a visual cursor (e.g., Honda et al., 2013; Takamuku and Gomi, 2015).

Previous studies suggest that prediction error—the discrepancy between the predicted and actual sensory outcomes of an action—can induce pseudo-haptic sensations. Prediction error is continuously computed and updated during an action as the system monitors incoming sensory feedback. Manipulations of the C/D ratio as well as delays in visual feedback can generate such prediction errors because they introduce temporal and/or spatial discrepancies relative to the predicted outcome. Honda et al. (2013) reported that, in a reaching task in which a cursor was controlled using a manipulandum, delayed visual feedback of the cursor evoked a pseudo-haptic sensation of heaviness. Moreover, adaptation to this delay reduced both the perceived heaviness and the sensitivity to the delay. These findings indicate that reducing prediction error through adaptation weakens pseudo-haptic sensations, suggesting that prediction error plays a role in modulating them. In addition, several previous studies have shown that pseudo-haptic sensations can be comparable to actual haptic stimulation and can influence the perception of physical stimuli in weight perception (Dominjon et al., 2005; Taima et al., 2014; Samad et al., 2019). Taken together, both temporal (i.e., delay) and spatial (i.e., displacement) factors appear to influence pseudo-haptic sensations at the sensorimotor level.

On the other hand, Takamuku and Gomi (2015) argued that prediction error may not be directly responsible for pseudo-haptic sensations. In their study, they manipulated the delay of a cursor controlled with a stylus pen and measured the resulting resistive sensation. In the task, participants were required to move the cursor back and forth between horizontally arranged targets at specified cycles, while the cursor movement was delayed relative to their actual input. The authors found that introducing a delay in the cursor movement produced a distinctive resistive sensation. However, the positional prediction error did not correlate with the magnitude of this sensation. Instead, they reported that a force cue—operationalized as the acceleration of the cursor—was positively correlated with the perceived resistance. Based on this result, they suggested that the sensation was influenced by a force implicitly inferred from the speed of the visual motion. In other words, the discrepancy between control and display may be resolved by interpreting the distorted visual feedback as if a mechanical load were acting on the movement.

Furthermore, Yokosaka and Kawabe (2022) proposed that pseudo-haptic sensations can be evoked purely by visual information that is unrelated to the C/D ratio and are determined according to a statistical internal model of the external world. They demonstrated that when the luminance of a square displayed on the screen was changed by a key press, delays or slower changes in luminance induced sensations of heaviness and stiffness, but not bumpiness, during luminance control. In this task, the key press and the luminance change were arbitrarily linked and had no direct relationship with prediction error. Nevertheless, sensations of heaviness and stiffness were induced when the luminance change controlled by the key press was delayed or slowed. These results suggest that observers rely on statistical internal models of the external world—for example, that slower motion is a diagnostic feature of heavier or stiffer objects—and that pseudo-haptic sensations arise when such models are selected based on visual information. Similarly, Kawabe et al. (2021) showed that reducing the speed of a cursor whose movement was initiated by a key press induced a sense of resistance. Importantly, participants were not asked to report their sensations using terms referring to specific sensory modalities, resulting in reports of multimodal experiences (Kawabe et al., 2021; Yokosaka and Kawabe, 2022). Taken together, these findings suggest that distorted visual information activates beliefs derived from statistical association that heavier objects move or start moving more slowly, thereby evoking pseudo-haptic sensations through multimodal integration. From this perspective, prediction errors are considered merely by-products of distorted visual feedback and are not directly responsible for pseudo-haptic sensations.

In the present study, we further investigated the mechanisms underlying pseudo-haptic sensations by separating the effects of prediction error from movement speed that may activate beliefs based on the statistical relationship between weight and movement. We designed a condition that produced spatial prediction errors by manipulating the control ratio of the cursor's movement direction without altering its movement magnitude. Specifically, participants moved the cursor toward a target along a displayed pathway while the cursor movement was blended with pre-recorded motion trajectories to manipulate spatial prediction errors (i.e., angular errors) (Wen et al., 2018, 2020; Wen and Haggard, 2020). This manipulation affected prediction errors in the spatial domain only, leaving the moving speed unchanged. Furthermore, the cursor's movement speed was reduced within a specific region near the center of the pathway to manipulate the C/D ratio. This design manipulated movement speed independently of spatial prediction errors. After each trial of the reaching task, participants rated their perceived sense of resistance. Moreover, because speed reduction also produces prediction errors during the movement, we asked participants to rate their sense of agency over the cursor, which reflects the amount of prediction errors perceived.

Using this design, we can disentangle the effects of spatial prediction errors and movement speed on pseudo-haptic sensations. Specifically, spatial prediction errors can be calculated from the angular difference between one's mouse movement direction and the displayed movement direction of the cursor. In contrast, movement speed can be quantified by other aspects such as the time required to reach the end of the pathway. The contributions of these two indices to the sense of resistance can then be analyzed. Furthermore, the measure of the sense of agency provides an indirect index of *perceived* prediction errors (Wen et al., 2015; Chang and Wen, 2026). Therefore, this approach also allows us to examine how the relationship between the sense of resistance and the sense of agency may differ under the manipulation.

Additionally, in the present study, we focused on pseudo-haptic resistance (Takamuku and Gomi, 2015; Kawabe et al., 2021) rather than friction or other haptic properties while participants controlled a cursor using a mouse. This is because “resistance” is a more natural description of the experience in a task where participants move a mouse on a smooth mouse pad without touching any object other than the mouse itself. Previous studies have reported that acceleration (Takamuku and Gomi, 2015) and movement speed (Kawabe et al., 2021) are critical factors in producing both a sense of resistance and sense of friction (Lecuyer et al., 2000; Ujitoko et al., 2019) which are linked to pseudo-haptic sensations.

## 2 Materials and methods

### 2.1 Participants

Twenty-three adults (8 males and 15 females; aged 18–31 years) participated in the experiment. All participants except one were right-handed. The left-handed participant uses their left hand to operate the mouse in daily life. All participants used their dominant hand to operate the mouse. All reported normal or corrected-to-normal vision and normal tactile sensation. The study was approved by the local ethics committee of Rikkyo University (reference number: 23–40). All participants provided written informed consent prior to participating in the study. The research was conducted in accordance with the ethical principles of the World Medical Association Declaration of Helsinki.

The required sample size was estimated using G^*^Power (Faul et al., 2007; Erdfelder et al., 2009) based on pilot testing data (*N* = 3). The analysis aimed to determine the sample size required to detect whether spatial prediction error induces pseudo-haptic sensations. The estimation focused on the comparison between the non-manipulated condition (i.e., speed ratio = 1.0; control ratio = 1.0) and the condition with the smallest reduction in control ratio without speed reduction (i.e., speed ratio = 1.0; control ratio = 0.75) within a repeated-measures ANOVA with a 3 (speed ratio) × 3 (control ratio) factorial design (see Procedures for details). Because multiple comparisons were planned (36 possible pairs in total), we adopted a conservative adjusted significance level corresponding to the smallest alpha (α = 0.05/36) in the Holm–Bonferroni correction procedure. An *a priori* power analysis for a two-tailed paired-samples *t*-test was then conducted using an effect size of Cohen's dz = 0.99, the adjusted alpha level (α = 0.05/36), and a power of 0.80. The analysis indicated that a minimum of 22 participants would be required.

### 2.2 Apparatus and stimuli

The experiment and all stimuli were controlled using a PC (HP ENVY Desktop TE02-1xxx) running Python with PsychoPy (Peirce, 2007, 2008; Peirce et al., 2019).

**Figure 1:**
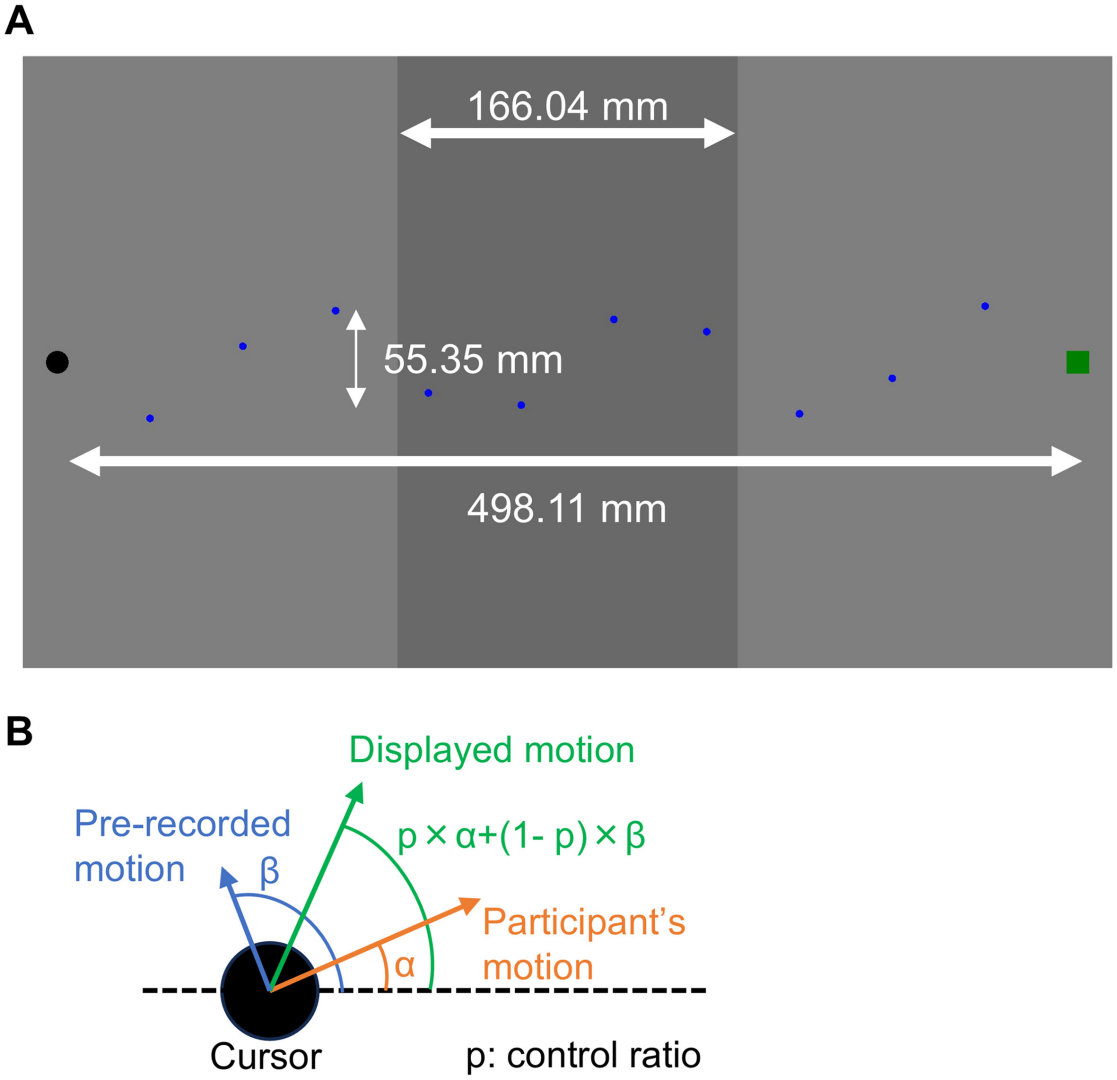
Schematic illustration of the visual stimuli. **(A)** Visual stimuli presented on the display. The black dot indicates the cursor controlled by participants, and the green square indicates the target. The blue dots represent the pathway guide. Cursor speed was reduced within the dark gray area at the center of the display. The trial ended when the cursor touched the target. **(B)** Method of blending pre-recorded motion with participants' motion. Only the direction of movement was averaged according to the control ratio, while the movement distance was determined by the participants' own motion.

Visual stimuli were presented on a 24-inch display (Dell P2424HT; 1,920 × 1,080 pixels; 60 Hz) with a gray background. A black dot served as the cursor, with a radius of 2.78 mm (0.27°). The cursor was controlled using a mouse (Logicool MX1800GR) on a mouse pad (HyperX HX-MPFS-XL; 900 mm × 420 mm). The target was a green square measuring 11.07 mm (1.06°) in width. In addition, ten blue dots, each with a radius of 0.93 mm (0.09°), were presented to guide participants' cursor trajectories. These dots were positioned between the cursor's starting position and the target along a sine wave with three cycles, an amplitude of 55.35 mm (5.28°), and a width of 498.11 mm (47.23°). The central region of the display was filled with dim gray and had a width of 166.04 mm (15.76°) (Figure 1A). The presentation of a sine-wave shaped pathway was used to prevent participants from making straight horizontal movements, which might rely more on feedforward control than on feedback control. In addition, deviations from the instructed pathway were not strictly prohibited.

The pre-recorded motion used in the present study was originally created for a target detection task in previous studies on the sense of agency (Wen and Haggard, 2018, 2020; Wen et al., 2020). In those tasks, participants were instructed to move a cursor using a mouse while both a target and several distractors moved on the screen. The movement direction of the distractors was morphed from the participant's mouse input and the pre-recorded motion at a 50/50 ratio, whereas the target's direction was morphed at a different ratio. Participants were then required to detect the target among the distractors. The pre-recorded motion itself was created by recording the movements of other participants performing similar tasks and contained approximately 50,000 frames of motion data. Consequently, the pre-recorded motion was entirely unrelated to the task in the present study, and participants could not predict its movements.

### 2.3 Procedures

Participants sat in front of the display at a viewing distance of approximately 60 cm. They were instructed to use the mouse to move the cursor to the target while following the blue dots arranged along the sine wave as closely as possible. However, even when the cursor trajectory substantially deviated from the pathway, the task was not stopped and the trial was not excluded from the analysis because the pathway served only as a guide and participants' movements were not restricted. The task continued until the cursor reached the target, and no time limit was imposed.

At the beginning of each trial, the cursor was presented on the left side of the display, 249.05 mm (23.45°) from the center. Participants were instructed to place their hand on the left side of the mouse pad before starting the task. During the reaching movement, they were instructed not to lift the mouse from the mouse pad and not to touch the mouse pad directly with their hand in order to eliminate additional haptic cues. When the cursor entered the dim gray area of the display, its speed was reduced to one of three levels: 1.0, 0.5, or 0.25 times the normal speed. We also manipulated the control ratio of the cursor for each participant. Specifically, the movement angle of the cursor was computed by combining the participant's movement with pre-recorded motion that was irrelevant to the reaching task, whereas the movement distance strictly followed the participant's own movement. The starting frame of the pre-recorded motion was randomly assigned on each trial, and the sequence was reset to the first frame when it reached the end. Three control-ratio conditions were implemented: 0.5, 0.75, and 1.0, corresponding to the proportion of the participant's movement in the angular component (Figure 1B).

After the cursor reached the target, participants completed questionnaires assessing the sense of resistance and the sense of agency. Responses were provided using a 0–100 slider scale with an anchor width of 166.04 mm (15.76°). For the measure of resistance, participants rated the statement:

“How much resistance did you feel on your hand while the cursor was in the dim gray area? (0 = no resistance at all; 100 = very strong resistance).

For the measure of the sense of agency, participants rated the statement:

“How much did you feel that you could control the cursor as you intended?” (0 = “I was completely unable to control the cursor”; 100 = “I was completely able to control the cursor”).

The experiment consisted of nine practice trials (one trial for each condition) and 90 main trials (ten trials for each condition). The order of trials was randomized for each participant. All participants were able to complete every trial and reach the target even under low-control conditions. Therefore, no trials or participants were excluded from the analysis.

### 2.4 Statistical analyses

First, we conducted 3 (speed ratio: 1.0, 0.5, and 0.25) × 3 (control ratio: 0.5, 0.75, and 1.0) repeated-measures analysis of variance (ANOVAs) on ratings of the sense of resistance and the sense of agency to examine the overall effects of the manipulated factors on the independent variables. Mauchly's test of sphericity was performed for all main effects and interactions. When the test indicated a violation of sphericity (*p* < 0.05), the Greenhouse–Geisser correction was applied. The results of Mauchly's test revealed violations of sphericity for both main effects in the analysis of the sense of agency, as well as for both main effects and their interaction in the analysis of the sense of resistance. When the ANOVA revealed a significant main effect or interaction, *post hoc* multiple comparisons were conducted using the Holm–Bonferroni correction to adjust the alpha level.

Next, the correlations and partial correlations (while controlling the effects of control ratio and speed ratio) were computed between the senses of resistance and time required to complete each trial for each individual. This analysis was used to examine whether the moving speed of the cursor along the pathway (i.e., the faster, the earlier to reach the end of the pathway) was a cue for the sense of resistance. The average of the correlation coefficients was then compared to zero using one-sample *t*-tests.

Finally, a linear mixed-effects model (LMM) was used to analyze the data. The averaged angular difference between the actual mouse movement and the displayed cursor movement in each trial, and the time required to complete each trial, and their interaction were included as fixed effects. Participant was included as a random intercept to account for individual differences. The first fixed effect corresponded to spatial prediction error, whereas the second corresponded to effective movement speed. This approach allowed us to retain trial-level observations while controlling for the non-independence of measurements within participants. All analyses were performed using JASP (JASP Team, 2025), except for the calculation of correlations and partial correlations, which were conducted in Python using pingouin (Vallat, 2018).

## 3 Results

### 3.1 Manipulation check

**Figure 2:**
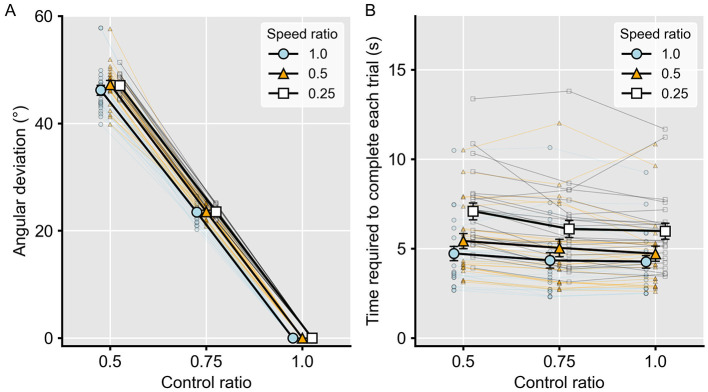
Data from the manipulation check. **(A)** Cursor deviation from the actual input against each control ratio. Cursor deviation was defined as the difference between the participant's intended cursor direction (i.e., the actual input) and the displayed cursor direction (i.e., the distorted visual feedback). **(B)** Time required to complete each trial in each condition. In all panels, different lines indicate speed ratios, the x-axis represents the control ratio, and error bars denote standard errors of the mean. Faint points connected by thin lines represent individual participants' data.

Figure 2A shows the angular deviation between participants' movement direction and the cursor's movement direction within the manipulated area for each condition. The main effect of control ratio was significant [(*F*_(1.13, 24.95)_ = 5368.10, *p* < 0.001, η_p_^2^ = 1.00)], whereas the main effect of speed ratio and the interaction between control ratio and speed ratio were not significant [(*F*_(2, 44)_ = 0.98, *p* = 0.39, η_p_^2^ = 0.04]; [*F*_(2.04, 7.10)_ = 0.60, *p* = 0.56, η_p_^2^ = 0.03], respectively). These results confirm that only the control ratio manipulation produced spatial prediction errors.

In contrast, Figure 2B shows the time required to complete each trial in each condition. The main effect of control ratio [(*F*_(1.46, 32.15)_ = 13.45, *p* < 0.001, η_p_^2^ = 0.38)], the main effect of speed ratio [(*F*_(2, 44)_ = 175.96, *p* < 0.001, η_p_^2^ = 0.89), and the interaction between control ratio and speed ratio [(*F*_(2.68, 58.85)_ = 6.21, *p* < 0.001, η_p_^2^ = 0.22)] were all significant. These results indicate that both speed reduction and control ratio increased the time required to complete a trial, with speed reduction showing a larger effect size. Although the control ratio did not affect the absolute speed of the cursor, it caused the cursor to deviate from the intended path, thereby effectively reducing the movement speed along the instructed pathway.

### 3.2 Sense of resistance

**Figure 3:**
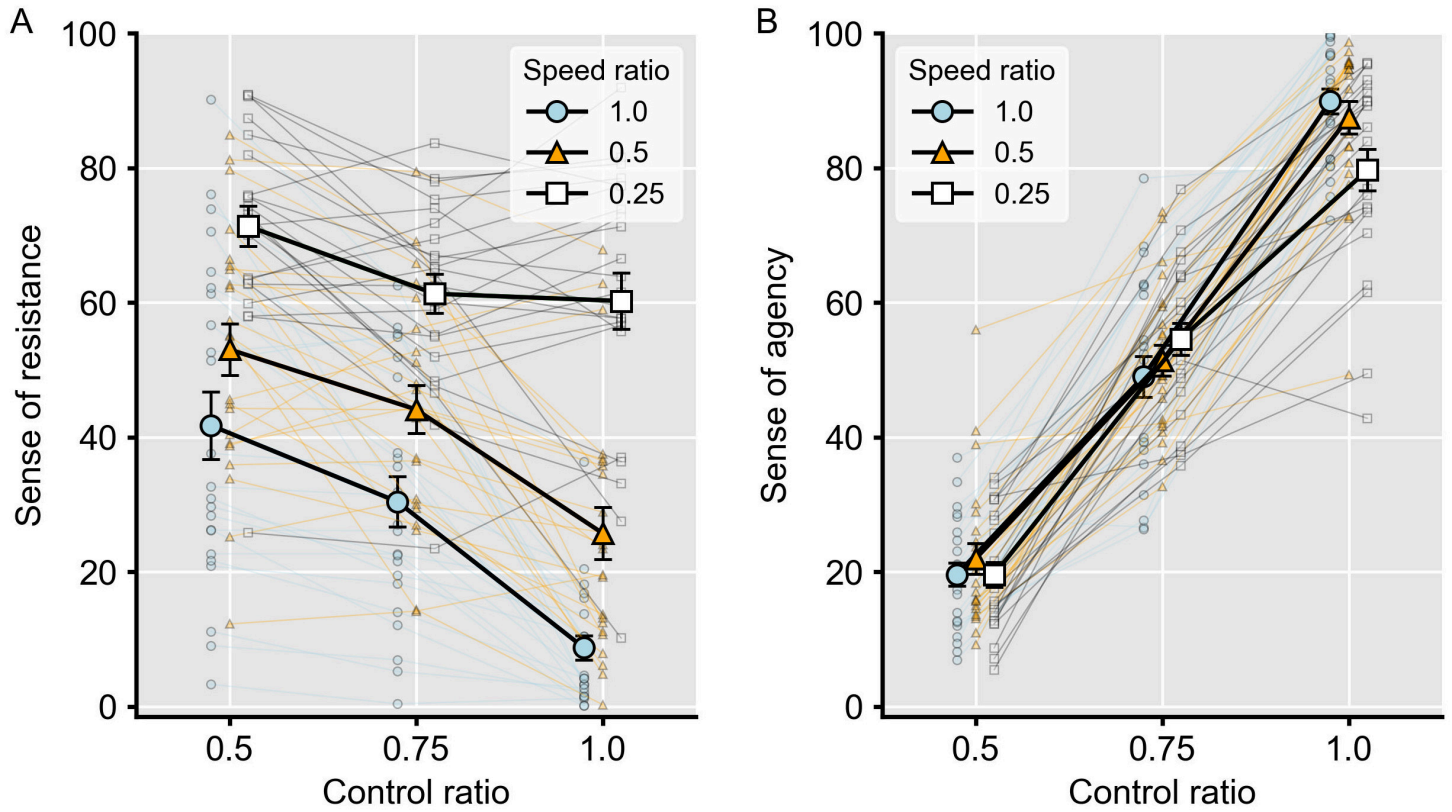
Results of the experiment. **(A)** Scores of the sense of resistance. **(B)** Scores of the sense of agency. In all panels, different lines indicate speed ratios, the x-axis represents the control ratio, and error bars denote standard errors of the mean. Faint points connected by thin lines represent individual participants' scores.

Figure 3A shows the sense of resistance in each condition. The ANOVA revealed significant main effects of speed ratio [(*F*_(1.39, 30.51)_ = 73.72, *p* < 0.001, η_p_^2^ = 0.54)] and control ratio [(*F*_(1.21, 26.55)_ = 25.44, *p* < 0.001, η_p_^2^ = 0.77)], as well as a significant interaction between the two factors [(*F*_(4, 88)_ = 17.58, *p* < 0.001, η_p_^2^ = 0.44)]. *Post hoc* multiple comparisons showed that all comparisons across speed ratios within the same control ratio were significant, with lower speed ratios producing greater perceived resistance (*t*s > 3.15, *p*s < 0.02, Cohen's *d*s > 0.65). For control ratio, when the speed ratio was hold constant, the comparisons between 0.75 and 0.5 at a speed ratio of 0.25 (*t*(22) = 2.64, *p* = 0.07, Cohen's *d* = 0.57), between1.0 and 0.75 at 0.25 (*t*(22) = 0.28, *p* = 1.00, Cohen's *d* = 0.06), and between 0.75 and 0.5 at 0.5 (*t*(22) = 2.35, *p* = 0.13, Cohen's *d* = 0.51) did not reach significance. However, all other comparisons within the same speed ratio revealed significant differences, with lower control ratios producing greater resistance (*t*s > 2.29, *p*s < 0.04, Cohen's *d*s > 0.64). These results indicate that both the speed ratio and the control ratio significantly influenced the sense of resistance, and that the effect of the control ratio varied depending on the level of speed ratio.

### 3.3 Sense of agency

As for the sense of agency (Figure 3B), the ANOVA revealed a significant main effect of control ratio [(*F*_(1.50, 33.05)_ = 305.69, *p* < 0.001, η_p_^2^ = 0.93)] and a significant interaction between control ratio and speed ratio [(*F*_(2.84, 62.49)_ = 9.94, *p* < 0.001, η_p_^2^ = 0.31)], but no significant main effect of speed ratio [(*F*_(1.40, 30.79)_ = 1.09, *p* = 0.33, η_p_^2^ = 0.05)]. *Post hoc* multiple comparisons showed that, when the control ratio was 1.0, the sense of agency at a speed ratio of 0.25 was lower than that at 1.0 (*t*(22) = 4.65, *p* < 0.001, Cohen's *d* = 0.90) and 0.5 (*t*(22) = 3.55, *p* < 0.01, Cohen's *d* = 0.69). However, no other comparisons for speed ratio reached significance (*t*s < 2.54, *p*s > 0.09, Cohen's *d*s < 0.49). In contrast, for control ratio, all comparisons at the same speed ratio revealed significant differences, with higher control ratios producing a greater sense of agency (*t*s > 8.24, *p*s < 0.001, Cohen's *d*s > 2.21). These results indicate that although a large reduction in speed weakened the sense of agency when participants had full control of the cursor, speed changes did not affect the sense of agency overall. In contrast, the control ratio strongly influenced the sense of agency.

### 3.4 Correlations between trial time and sense of resistance

**Figure 4:**
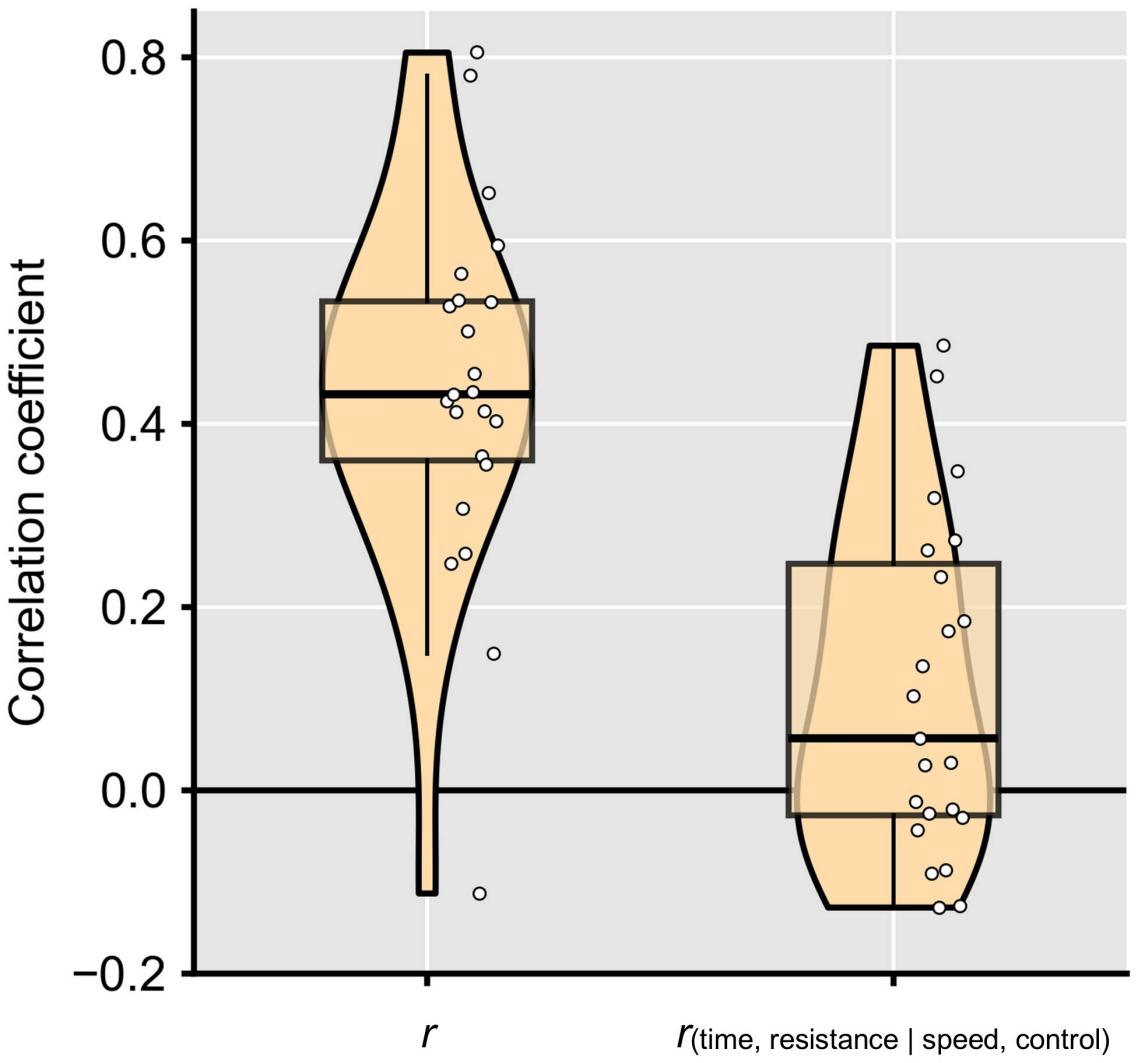
Correlation and partial correlation coefficients between the time required to complete each trial and the sense of resistance. Partial correlations were computed while controlling for speed ratio and control ratio for each participant. The violin plots represent the kernel density distributions of the data, while the boxplots indicate the interquartile range with the median marked by a horizontal line. Whiskers extend to 1.5 × the interquartile range. Each dot corresponds to one participant.

We further calculated the correlation and partial correlation between the time required to complete each trial and the sense of resistance while controlling for speed ratio and control ratio for each participant (Figure 4). The average correlation coefficients were then compared to zero using a one-sample *t*-test. Both the correlation and the partial correlation between the time required to complete each trial and the sense of resistance were significantly larger than zero (mean of *r* = 0.44, *SD* = 0.20, *t*(22) = 10.61, *p* < 0.001, Cohen's *d* = 2.21; mean of *r*_(time, resistance|speed, control)_ = 0.11, *SD* = 0.18, *t*(22) = 2.870, *p* = 0.01, Cohen's *d* = 0.60). These results showed that participants were very likely to use the movement speed of the cursor as a cue for the sense of resistance, even when excluding the effects of the experimental manipulations.

### 3.5 The contribution of spatial prediction errors and moving speed on the sense of resistance

We fitted a LMM with spatial error (angular deviation) and trial completion time as fixed effects and participant as a random intercept. The results showed a significant positive effect of spatial error on the sense of resistance (*b* = 0.93, *SE* = 0.10, *t*(65.38) = 9.79, *p* < 0.001). Trial completion time also had a significant positive effect (*b* = 1.06, *SE* = 0.49, *t*(437.22) = 2.14, *p* = 0.03). The interaction between spatial error and time was not significant (*b* = 0.01, *SE* = 0.01, *t*(417.64) = 0.76, *p* = 0.45). The results indicated that both effective movement speed and spatial prediction error significantly contributed to the sense of resistance.

## 4 Discussion

In the present study, we aimed to investigate the mechanism of pseudo-haptic sensation. Previous studies have proposed two hypotheses: one is that prediction error induces pseudo-haptic sensations (Honda et al., 2013), and the other is that visual information regarding movement magnitude, which activates beliefs based on statistical models, modulates haptic sensation in a multimodal manner (Takamuku and Gomi, 2015; Kawabe et al., 2021; Yokosaka and Kawabe, 2022). It remained unclear whether either theory alone is sufficient, or whether both contribute to pseudo-haptic sensations through different processes. In our experiment, participants controlled a cursor using a mouse and were instructed to reach the target along the displayed pathway, and reported the sense of resistance that they felt in their hands. Our manipulation of control ratio only affected the movement direction of the cursor without affecting the movement magnitude of the visual stimulus. On the other hand, the manipulation of movement speed resulted in changes in both the information about movement magnitude and prediction errors (in both temporal and spatial domains). The results indicated that both spatial prediction error and object movement speed independently contributed to the sense of resistance.

First, the results showed that both the manipulation of control ratio and movement speed had significant effects on pseudo-haptic sensations. A larger speed reduction induced a stronger pseudo-haptic sensation. Notably, a lower control ratio also induced a greater sense of resistance even when there was no speed change. However, when we consider the movement of the visual stimulus in the *intended* direction (i.e., the instructed pathway), although the control ratio did not change the absolute movement magnitude, it had a significant effect on the time required to complete each trial. Specifically, when the control ratio was lower, participants took longer to reach the end of the pathway. This means that the *effective moving speed* along the pathway was actually lower. Furthermore, the correlation analyses revealed a significant correlation between the time required to complete each trial and the sense of resistance, even after excluding the effects of control ratio and speed reduction, indicating that the effective moving speed may indeed have been used as a cue for the sense of resistance. Taken together, the results support the possibility that the pseudo-haptic sensation is induced by the visual information regarding movement magnitude. This is consistent with the theory of statistical relationship between weight and moving speed (Takamuku and Gomi, 2015; Kawabe et al., 2021; Yokosaka and Kawabe, 2022). Furthermore, the results also suggest that when discussing the effect of movement magnitude on pseudo-haptic sensations, it is necessary to consider the *intended moving direction*. When it takes more effort to fulfill that intention, external physical factors such as resistance become a likely explanation derived from statistical models.

Furthermore, although the main effect of control ratio on the sense of resistance was significant, the control ratio also reduced the effective movement speed. Therefore, it remained unclear whether spatial prediction errors alone could induce the sense of resistance when controlling for the effect of movement speed. To address this issue, we conducted a linear mixed-effects model analysis. The results showed that both spatial prediction error and trial completion time had significant fixed effects, whereas their interaction was not significant. These results indicate that, in addition to effective movement speed, spatial prediction errors also contribute to the sense of resistance. In other words, even if the cursor moves equally quickly, greater deviation in its movement direction is associated with a stronger sense of resistance.

Finally, we also asked participants to rate their sense of agency over the cursor. We found that speed reduction affected the sense of agency only in the high-control condition, suggesting that speed reduction is a weaker cue for the sense of agency than spatial deviation. The pattern of agency ratings differed markedly from that of resistance ratings. If the sense of agency is considered a direct reflection of prediction errors at both the sensorimotor and intention levels (Wen et al., 2015; Chang and Wen, 2026), this would suggest that the sense of resistance is unlikely to arise directly from prediction errors alone. Instead, the sense of resistance is more likely to be induced by both predictive processes and the statistical relationship between weight and movement speed. These two factors may partially overlap in many situations. For example, large prediction errors often slow effective movement speed, and speed reductions can also produce spatial and temporal prediction errors, as shown in our experiment. Nevertheless, we suggest that these two factors likely contribute independently to pseudo-haptic sensations. That is, even when effective movement speed does not change, spatial deviations can still induce pseudo-haptic sensations, and vice versa.

Taken together, we suggest that both spatial prediction errors and the statistical relationship between weight and movement speed contribute to pseudo-haptic sensations. These two factors overlap in many situations, and either theory alone may appear sufficient to explain pseudo-haptic sensations in many tasks. However, our analyses revealed that even after controlling for one factor, the effect of the other remained significant. People can experience a sense of resistance not only when the object moves slowly, but also when it spatially deviates from the intended movement direction.

Several limitations of the present study should be acknowledged. First, the present study focused on the sense of resistance. Pseudo-haptic techniques are widely applied across different contexts and can generate various sensations, including kinematic properties (e.g., resistance, weight, compliance) as well as geometric properties such as bumps and holes (Lécuyer et al., 2004; Mensvoort et al., 2010). Therefore, it remains unclear whether the present results generalize to other devices, tasks, or types of pseudo-haptic sensations. Future studies should examine whether similar mechanisms operate across these different situations. Second, although the results showed that both spatial prediction errors and effective movement speed significantly contributed to the sense of resistance, it remained unclear whether the sense of resistance induced by these two factors represents qualitatively the same experience. Specifically, spatial prediction errors may produce a sense of resistance that feels “bumpy” or “rough”, whereas movement speed may be associated with a “heavier” feeling. Subjective ratings may lack the resolution to distinguish between these experiences. Future studies should consider including behavioral and/or physiological measures, in addition to subjective ratings, to address this issue more carefully. At last, both the internal model that generates prediction errors and the statistical model linking weight and movement speed may be updated through learning and adaptation. Future research should therefore examine how pseudo-haptic sensations change during learning and adaptation.

## Data Availability

The datasets presented in this study can be found in online repositories. The names of the repository/repositories and accession number(s) can be found below: Open Science Framework (OSF) [https://doi.org/10.17605/OSF.IO/54FZT].
